# Architecture and mental health wellbeing versus architecture therapy for mental disorders

**DOI:** 10.1192/j.eurpsy.2023.2106

**Published:** 2023-07-19

**Authors:** N. Bouayed Abdelmoula, E. Abdelmoula

**Affiliations:** 1 Genomics of Signalopathies at the service of Medicine, Medical University of Sfax, Sfax; 2 M2RCA, Ecole Doctorale Sciences et Ingénierie Architecturales (ED-SIA), Tunis, Tunisia

## Abstract

**Introduction:**

Architecture is a particular art as well as a transversal science that evolves according to multiple variables that call upon, aesthetics, sociology, political science, technology, history, cultures, economy, tourism, as well as the satisfaction of human needs and the physical/psychological health. It addresses the four senses of human and it humbly configures man’s environment, constitutes the set or the framework in which he evolves and in which he becomes an actor, both overwhelming and magical as Aldo Rossi says (1981).

**Objectives:**

We aim through this review to show how does architecture affect human mental health wellbeing and what can we do to create a therapeutic architecture.

**Methods:**

We comprehensively review the scientific literature using Pubmed database and other search platforms such as Google scholar to state the relationship between architecture and human mental health wellbeing and to delineate the meaning of architecture therapy in the field of mental disorders.

**Results:**

Our bibliographic research revealed that Architecture and mental health are directly linked and that the concept of therapeutic architecture or architectural therapy has been generated in 1984 by Roger Ulrich, Professor of Architecture at the Centre for Health Design in Sweden to designate the influence of the environment on the healing and recovery process of patients. In fact, Architecture has the ability to create meaningful spaces. Churches, mosques and cathedrals planned centuries ago are a testament to architectural wonders, as they evoke a sense of magnificence, royalty, glory, serenity and tranquility. The intriguing details of these spaces were meant to induce positive feelings and emotions and are considered architectural masterpieces to this day, as they continue to fascinate humans around the world. In the other hand, many studies showed the positive effect of nature (therapeutic horticulture) and exposure to the outdoors; towards distraction from stress and anxiety levels of patients in a Health care setup. Recent studies analyzed how Architecture and interior design (safety and security, noise and external stressors, space and interior layout, nature, lighting and atmosphere, art, community, etc…) are important not only for the well-being of patients in health care centers, but also for humans in general in their living environments.

**Conclusions:**

Mental health is impacted by different aspects of architecture and design. Incorporating elements such as natural lighting, open floor plans, private and open community spaces, artwork, safety procedures, and nature/views of nature, provides a supportive environment for the mental well-being and the treatment of mental disorders.

**Disclosure of Interest:**

None Declared

